# Exuberant liver adenomatosis presenting with iron deficiency anemia

**DOI:** 10.1002/ccr3.814

**Published:** 2017-03-16

**Authors:** Catarina Mota, Ana Margarida Carvalho, Válter Fonseca, Marisa Teixeira Silva, Rui M. M. Victorino

**Affiliations:** ^1^ Clínica Universitária de Medicina IICentro Hospitalar Lisboa NorteLisboaPortugal

**Keywords:** Hepatocellular adenoma, intratumoral hemorrhage, iron deficiency anemia, liver adenomatosis

## Abstract

Intratumoral or intraperitoneal hemorrhage is a recognized complication of liver adenomatosis. We report a case of multifocal massive liver adenomatosis presenting as chronic iron deficiency anemia. Clinicians’ awareness about this atypical presentation not highlighted in the literature is important to allow timely diagnosis and surgical intervention to prevent fatal complications.

## Introduction

Hepatocellular adenoma is an uncommon benign hepatic tumor, with an increased incidence in women of reproductive age with a history of oral contraceptives 1, 2, men taking anabolic steroids 3, or patients with glycogen storage disease or iron‐overload disorders 4, 5. The majority of hepatic adenomas are solitary, although the occasional occurrence of two or three adenomas is well recognized in the literature 6, 7.

Liver adenomatosis, defined by the presence of multiple adenomas (arbitrarily, defined by more than 10) in a normal hepatic parenchyma, was described in 1985 by Flejou et al. as a distinct entity 8. Subsequently, case reports and small series have documented multiple adenomas in an otherwise normal liver, both in men and women, in the absence of glycogen storage disease or association with steroid medication 9, 10, 11, 12. Nevertheless, the role of estrogen intake in the outcome and progression of liver adenomatosis is still controversial, in contrast to solitary adenomas where the association with estrogens is well established 13.

The pathogenesis and natural history of liver adenomatosis are still unclear. Some reports show that hepatic adenomatosis occurs more often in patients who have coexistent vascular tumors, portal vein absence, or occlusion or portohepatic venous shunts, leading to a speculative association between this entity and congenital or acquired abnormalities of the hepatic vasculature 14, 15, 16. Germline mutation of hepatocyte nuclear factor‐1 alpha, which is associated with maturity‐onset diabetes of the young type 3, has been also recently implicated in some reported cases 17, 18.

Although considered a benign entity, liver adenomatosis has been associated with an increased risk of malignant transformation and hemorrhage, both potentially fatal 8, 13. Complications of adenomatous lesions, such as intraperitoneal bleeding, intratumoral hemorrhage, or necrosis‐producing acute pain, often reveal a frequently silent disease 8, 9, 19, 20, 21, 22.

We describe a rare case of massive and multifocal liver adenomatosis, who presented clinically as a chronic iron deficiency anemia due to chronic intratumoral bleeding that illustrates an unreported presentation of this condition.

## Case Report

A 37‐year‐old woman with no relevant clinical background, on oral contraception with desogestrel/etinilestradiol, was admitted with fatigue on moderate exertion and anemia detected in routine laboratory tests.

Physical examination revealed pallor, with reasonable general condition, blood pressure 122/70 mmHg, pulse rate 95/min, and respiratory rate 16/min. There were no rashes or skin lesions nor palpable lymphadenopathy. Cardiovascular and pulmonary examination was normal. The abdomen was normal and the remaining examination unremarkable.

The blood tests revealed an iron deficiency anemia with hemoglobin of 9.0 g/dL, mean corpuscular volume of 67.7 fl, iron of 17.6 mg/dL, and ferritin of 17 ng/dL, normal transaminases, alkaline phosphatase of 250 U/L (reference range 45–129 U/L), gamma‐glutamyl transferase of 91 U/L (reference range <73 U/L), normal bilirubin, C‐reactive protein of 4.78 mg/dL (reference range <0.4 mg/dL), and sedimentation rate of 81 mm/h (reference range <12 mm/h).

**Figure 1:**
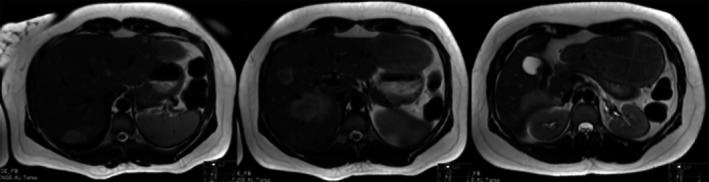
Abdominal CT: multiple hepatic adenomas.

Considering the differential diagnosis of iron deficiency anemia, menstrual blood loss was normal, with no evidence of menorrhagia. The patient was not vegetarian, with a diversified diet without restrictions. The endoscopy and the colonoscopy revealed no abnormalities. Abdominal ultrasound and CT scan showed hepatomegaly with bilateral focal lesions, namely a proliferative, solid lesion 12.5 × 12.3 × 6 cm contacting the gastric wall, a lesion occupying almost the entire segment VI (6 cm anteroposterior diameter), and a smaller lesion in the eighth segment with 20 mm (Fig. 1).

Multifocal hepatic lesions and iron deficiency anemia in a young woman under oral contraception favored a benign etiology, particularly hepatic adenomas, focal nodular hyperplasia, nodular regenerative hyperplasia, and hemangiomatosis, although focal liver lesions in malignant context had also to be considered.

Additional investigation revealed negative CEA, *α*‐fetoprotein, and CA 19.9, *β*2microglobuline of 2.48 mg/L, and negative viral serology. The abdominal magnetic resonance confirmed hepatomegaly with multiple focal lesions of solid nature, well above the limit of ten that defines liver adenomatosis, the larger in the left lobe. Biopsy of the major lesion showed marked sinusoidal ectasia, focal lymphocytic inflammatory infiltrate, ductular reaction, prominent vessels with thin wall, mild perivascular fibrosis, and positivity for GS‐6, beta‐catenin, and amyloid A, favoring the diagnosis of hepatocellular adenoma of inflammatory/telangiectasic type. The patient was diagnosed with hepatic adenomatosis and probable chronic intratumoral hemorrhage.

Atypical resection of segment III and enucleation of VI segment lesion with *Cavitron ultrasonic surgical aspirator* were performed. Histology of the resected lesions revealed hepatic adenomas with local steatosis, areas of hemorrhage and sinusoidal dilation and scarce ductular reaction. The patient was discharged after 6 days postoperatively, with the diagnosis of hepatic adenomatosis with intratumoral hemorrhage.

One month after surgery, the patient remained asymptomatic, with a gradual recovery in exercise tolerance. The blood tests revealed a progressive improvement of hemoglobin (10.2 g/dL), iron, and ferritin, with concomitant decrease in alkaline phosphatase and gamma‐glutamyl transferase. There was a marked reduction in inflammatory parameters, suggesting that they were in part attributed to the inflammatory syndrome associated with this particular histological type of adenomas, as previously described in the literature 23. Given the initial clinical presentation, the complementary investigation, and the observed evolution, iron deficiency anemia was interpreted as the inaugural presentation of liver adenomatosis as a result of chronic intratumoral hemorrhage.

## Discussion

Liver adenomatosis, defined as at least 10 adenomas in an otherwise normal liver, has been historically associated with an increased risk of malignant transformation and hemorrhage 8. Two forms have been described in the literature: the massive form, with liver enlargement and unilobar commitment, and the multifocal form, where the liver is not enlarged and one or two of the disseminated adenomas may be larger and produce complications. In the previous reported cases of liver adenomatosis, the diagnosis was made following complications of adenomas – intraperitoneal bleeding 8, 9, 19, intratumoral hemorrhage or necrosis determining acute pain 15, 20, 21, 22, symptomatic or asymptomatic hepatomegaly 8, 24 or as an incidental discovery. The therapeutic management of this entity ranges from conservative monitoring or medical therapy to aggressive surgery and even orthotopic liver transplantation, according to the size, number, localization, and predictable complications of the tumors 8, 9, 12, 18.

Spontaneous hemorrhage has been recognized as the most common complication of liver adenomatosis, particularly with large and subcapsular adenomas 12. The histologic features of liver adenomas, with a proliferation of hepatocytes and sinusoids and weak connective tissue support, predispose them to hemorrhage, particularly because these lesions are perfused almost exclusively by high‐pressure arterial flow 12. The exact frequency of hemorrhage is uncertain, because asymptomatic patients usually do not seek medical attention. Dokmak et al. reported that hemorrhage (as identified on imaging) was present in 21% of 122 patients with single or multiple hepatocellular adenomas and was related to the size of the tumor 2. In a small series of eight patients, Chiche et al. reported that the disease was revealed by intraperitoneal bleeding in two patients. One died before laparotomy, the diagnosis was made at necropsy, and the other underwent urgent surgery 13. Additionally, three patients were admitted for acute abdominal pain corresponding to intratumoral bleeding or necrosis of one of the adenomas 13. These findings emphasize the risk of hemorrhage as a major concern of this benign entity.

Notwithstanding the well‐recognized possibility of intratumoral or intraperitoneal hemorrhage, there are no reports of iron deficiency anemia as sole presentation of multifocal and massive hepatic adenomatosis. This case illustrates a rare unreported form of massive and multifocal liver adenomatosis clinical presentation that should be considered as the early diagnosis and surgical intervention can prevent possible fatal consequences of a benign entity.

## Authorship

CM: involved in conception of the work, data collection, data analysis and interpretation, drafting the article, critical revision of the article. AMC: involved in data collection, data analysis and interpretation, drafting the article, critical revision of the article. VF: involved in data collection, data analysis and interpretation, drafting the article, critical revision of the article. MTS: involved in data analysis and interpretation, drafting the article, critical revision of the article. RMMV: involved in data analysis and interpretation, drafting the article, critical revision of the article.

## Conflict of Interest

The authors declare that they have no conflict of interests.
